# Kinetic Monte Carlo Modeling of the Spontaneous Deposition of Platinum on *Au*(111) Surfaces

**DOI:** 10.3390/e27060619

**Published:** 2025-06-11

**Authors:** María Cecilia Gimenez, Oscar A. Oviedo, Ezequiel P. M. Leiva

**Affiliations:** 1 Instituto de Física Enrique Gaviola (IFEG), CONICET, Facultad de Matemática, Astronomía, Física y Computación (FaMAFyC), Universidad Nacional de Córdoba, Córdoba C.P. 5000, Argentina; 2 Laboratorio de Energías Sustentables, Córdoba C.P. 5000, Argentina; ezequiel.leiva@unc.edu.ar; 3 Instituto de Investigaciones en Físico-Química de Córdoba (INFIQC), CONICET, Departamento de Química Teórica y Computacional, Facultad de Ciencias Químicas, Universidad Nacional de Córdoba, Córdoba C.P. 5000, Argentina; o.a.oviedo@unc.edu.ar

**Keywords:** kinetic Monte Carlo simulations, platinum, gold surface, spontaneous deposition, adsorption, diffusion

## Abstract

The spontaneous deposition of platinum (Pt) atoms on Au(111) surfaces is systematically investigated through kinetic Monte Carlo simulations within the Embedded Atom Model framework. The kinetic model aims to capture both stoichiometric, atomic-scale interactions and the more relevant processes that describe the kinetics of a physical problem. Various deposition rates are examined, encompassing a thorough exploration of Pt adsorption up to a coverage degree of θ=0.25. The resulting 2D islands exhibit a ramified structure, mirroring the experimental methodologies. For the first time, this study extensively analyzes the dependence of both the mean island size and island density on spontaneous deposition, thereby offering valuable insights into the intricate dynamics of the system.

## 1. Introduction

When the size of a metallic material decreases to the nanometric scale, it shows unique properties that cannot be observed in macroscopic-sized materials. The number of synthesis methods for these nanomaterials and their new and possible technological applications has increased over the last decade. For a long time, metallic ultra-thin overlayers, consisting of a two-dimensional structure of monoatomic height, have been considered the finest state of manipulation [1,2]. Nowadays, it is possible to obtain metallic ultra-thin structures (small 2D clusters) with diameters of a few nanometers in a quick and reproducible way [3,4]. The methodology used for this is the electrochemical deposition of foreign metals on another metal and/or semiconductor substrate. Several elementary steps, such as electron transfer, desolvation, and diffusion, are involved in this process, which in many cases implies the formation of a new phase through a nucleation and growth process. Understanding these phenomena would allow for a more efficient design of these compounds.

Platinum is one of the most commonly used metals because it catalyzes a large number of chemical and electrochemical reactions [5,6,7]. This metal is not an abundant element in nature, and consequently, it is expensive. So, much effort has been invested in making efficient catalytic surfaces to minimize the amount of Pt used. Its deposition on less expensive, more abundant metals has been one of the strategies used to save Pt. However, in general, the properties of ultra-thin overlayers differ from those of bulk ones due to diverse structural and quantum effects dominating their properties [1,2,3,4]. Fortunately, the electrocatalytic properties of a substrate are often greatly improved by the deposition of a monolayer of Pt on its surface [3,4,8]. However, the size-dependent strain of Pt nanoclusters has some effect on the energy of the d-band center and therefore on the activity of Pt [9]. These facts show that it is necessary to deepen the understanding of the phenomena that control the morphology and size of the 2D clusters usually involved in nucleation and growth.

Since the interaction energy among Pt atoms is stronger than the interaction between Pt and other metal atoms, a three-dimensional growth or “Volmer–Weber” mechanism is expected under overpotential deposition (OPD) conditions [1,2,3,10,11]. Within this simplified picture of this electrochemical system, it appears, a priori, very difficult to produce ultra-thin films of Pt on other less noble metals. However, a lot of experimental work has shown that this is possible, so the simplified view given above appears to be too simple to depict the actual electrochemical context [3]. Anions and cations dissolved in the electrolyte, pH, solubility, temperature, and other factors can play a key role in the growth of ultra-thin-film phases. Furthermore, the electrochemical environment offers great advantages due to the ability to easily create and manage very large electric fields at the interfaces. All these factors allow for the stabilization of certain structures and the modification of others in a way that is impossible under ultra-vacuum conditions.

The deposition of ultra-thin films of Pt onto other less noble metals can be achieved through [12] electrochemical deposition (ED), spontaneous deposition (SD), and galvanic replacement (GR), also known as surface-limited redox replacement (SLRR). ED involves strict control of the deposition potential of the substrate on which the deposition occurs. Basically, a potential scan or step is applied to the substrate, which provides the electrons for the depositing ions, and a current transient occurs as a consequence of these events. The second method, SD, involves a first step, where the metal substrate is immersed in an electrolyte solution of a Pt-containing electrolyte (i.e., ${\left(PtCl_{6}\right)}^{2-}$), and a second step, where an irreversible electrochemical reduction of Pt(IV) to Pt(0) takes place. The structures that may be obtained with this procedure range from 2D clusters and ultra-thin films to multilayers [13,14,15,16,17]. The third method, GR, also called SLRR, involves the replacement of a previously adsorbed (via underpotential deposition (UPD)) sacrificial monolayer [3,4,8,18,19]. This method involves two stages: an electrochemical reduction leading to a UPD-deposited metal layer, which is later oxidized by the metal cations of the noble metal being deposited. The coverage of these thin films is controlled by the stoichiometry of the redox replacement reaction, the structure, and the coverage of the UPD monolayer. Thus, it is possible to obtain Pt monolayers (or submonolayers) easily and quickly.

Regarding SD, Strbac et al. [16] showed that Pt can be deposited on Au(111) surfaces from ${\left(PtCl_{6}\right)}^{2-}$. During the electrochemical reduction in the second step of SD, Pt islands were formed homogeneously on Au(111) via a nucleation and growth process, yielding nanosized Pt islands with no preference for steps or other surface defects. However, Waibel et al. [10] showed through STM that Pt deposition from ${\left(PtCl_{6}\right)}^{2-}$ or ${\left(PtCl_{4}\right)}^{2-}$ on Au(111) or Au(100) starts mainly at defects like step edges, and then three-dimensional clusters are formed. These are formed in different structures, such as $\left(2\sqrt{19}\times 2\sqrt{19}\right)R23^{\circ }$ [20], $\left(\sqrt{7}\times \sqrt{7}\right)R19.1^{\circ }$ [10,20], $\left(\sqrt{3}\times \sqrt{3}\right)R30^{\circ }$ [17], or (1×1) [16] on Au(111), as well as $\left(3\times \sqrt{10}\right)$ on Au(100) [10], in a broad range of cluster sizes and layer numbers. Similar behavior has been observed using other electrolytes, such as ${\left(PtBr_{4}\right)}^{2-}$ and ${\left(PtI_{6}\right)}^{2-}$ [20].

Brankovic proposed that Pt SD on Ru is the result of two coupled half-cell reactions,

(1) $${\left(PtCl_{6}\right)}^{2-}+4e^{-}\to Pt^{0}+6Cl^{-}$$

and

(2) $$Ru^{0}+xH_{2}O\to RuO_{x}H_{y}+\left(2x-y\right)H^{+}+\left(2x-y\right)e^{-}$$

where the deposition mechanism can be proposed based on the fact that the reaction takes place on an oxide-free Ru surface [4,21]. However, in the case of Pt SD on a Au surface, this cannot be assured. The potential for surface oxide formation on Au surfaces is more positive than the redox Pt deposition potential, so oxide formation can interfere with the SD reaction.

SD has also been applied using the UPD of hydrogen preadsorbed onto a flat surface [22] of Au [23] and on Pd [22] nanoparticles, leading to the formation of an ultra-thin Pt layer. More recently, Dai and Chen [24] showed that the SD approach can also be applied to achieve the growth of a Pt atomic shell on Au nanoparticles using ${\left(PtCl_{6}\right)}^{2-}$. In the latter work, the deposited Pt atoms were uniformly distributed on the nanoparticles, with the coverage tunable by the electrolyte concentration and temperature. Similarly, Kim et al. [25] showed that when the Pt-ion concentration is in the range $10^{-5}$–$10^{-4}$ M, the Pt deposits are nanoislands of monatomic height. In the concentration range $10^{-4}$–$10^{-3}$ M, the Pt deposits present mostly two-layer-thick nanofeatures. For higher concentrations, the deposits become wider and thicker. Pt 2D clusters on Au-NPs with tunable coverage can also be designed on multiwall carbon nanotubes [26]. Therefore, SD can be used to generate various types of Pt clusters and thin films on Au.

The ability to tune the size of 2D nanoclusters and/or the thickness of ultra-thin films requires a precise quantitative understanding of the phenomena that take place at the atomic level. An alternative to experimental work for accessing this information is the application of computational methods on the atomistic scale to simulate the surface reactions and transport phenomena of these electrochemical systems. Several numerical methods have been developed to simulate the formation of 2D nanoclusters or ultra-thin films. In general, two types of approaches have been used to simulate metal growth at the nanoscale: those that provide thermodynamic information, such as Monte Carlo (MC) or Grand Canonical Monte Carlo (GCMC) [27,28,29,30,31,32], and those that provide dynamic information, such as Molecular Dynamics (MD) or Kinetic Monte Carlo (KMC) [33,34,35,36,37,38,39,40]. MC and GCMC involve conducting numerical simulations using the introduction of random variables, whose values are assigned via the generation of random numbers. These methods provide information at the microscopic level for systems close to equilibrium, which may be converted into macroscopic information using concepts stemming from statistical mechanics (pressure, internal energy, etc.). MD is a deterministic approach that solves Newton’s equations (or some equivalent, depending on the assembly. To discretize time, time steps of the order of the femtosecond are required, which results in the dynamic behavior of the system being described on the nanosecond scale. However, many processes, especially those occurring in 2D nanocluster growth, take place on much larger time scales that are inaccessible through MD. For example, the experimental measurement of the activation energy for the diffusion of Au and Pt atoms performed by Alonso et al. [41] resulted in values of 0.607eV and 0.824eV, respectively. Assuming a constant frequency factor on the order of $10^{12}$ s^−1^ at 300K, within absolute velocity theory, diffusional times can be estimated to be on the order of seconds for Au and on the order of hours for Pt. Thus, MD does not appear to be an alternative to simulate the present phenomena.

On the other hand, the KMC technique [42] is stochastic in nature, and it is an efficient method for simulating physical phenomena that can be described in terms of Poisson processes. The theoretical foundation of the KMC technique was explained by Fichthorn in [43,44]. It is worth mentioning that with this technique, it is possible to reach experimental time scales, that is, on the order of seconds (or more). In Section 2, we will return to this topic. The KMC technique was employed by Timothy and Rikvold [37] to analyze metal deposition on a substrate seeded with 10-atom metal clusters. The authors analyzed the size distribution of 2D nanoclusters as a function of the energy barriers for surface diffusion, which ranged from 0.15 eV to 0.34 eV. Their results suggest that an energy barrier lower than 0.22 eV would produce a uniform size distribution of the clusters. Effective lateral interactions were also employed by Zhang et al. [27] to successfully reproduce experimental transients originating from the adsorption of cations and sulfate anions. A more realistic approach to the metallic deposition of bidimensional layers, also using KMC simulations, was taken in subsequent work by Gimenez et al. [33,34]. In that work, embedded-atom method (EAM) potentials were employed to emulate the interaction between particles in a metallic Ag-Au system. Also, Rafiee and Bashiri employed dynamic Monte Carlo simulations to study hydrogen production from formic acid decomposition on Ni(001) and Cu(100) [45,46]. They found the optimum temperature and pressure for hydrogen production and concluded that hydrogen is produced through direct dehydrogenation from formic acid.

Recent advances in KMC simulations led to the inclusion of atom-exchange phenomena. To account for collective surface diffusion processes, Treeratanaphitak et al. [39,40] included atom exchange and step-edge atom exchange in addition to nearest-neighbor hopping. This progress has led to a more realistic description of polycrystalline metal electrodeposition.

To the best of our knowledge, we have not detected work in the field of simulations devoted to understanding Pt nucleation and growth on Au surfaces. This system is the final state of many experimental routines, such as ED, SD, and GR, which have found increasing technological applications over the last decade, as mentioned above. The simulation of a wide range of deposit morphologies that arise from various combinations of reaction rate, transport, and geometric parameters represents a gap in the theoretical literature. Thus, the combination of EAM potentials with KMC appears to be a promising tool for studying the present system in order to correlate theoretical results with experimental results. With this purpose, the subject of the present study is the analysis of the characteristics of the spontaneous deposition (SD) of small 2D nanoclusters of Pt onto Au under different electrochemical conditions.

## 2. Model and Simulation Technique

### 2.1. General Model

The adsorption and diffusion of Pt atoms on Au(111) surfaces are modeled using kinetic Monte Carlo simulations. Surface alloys and multilayers are not allowed, since only one monolayer or submonolayer of Pt atoms is considered. Two kinds of adsorption sites can be occupied by Pt atoms: hollow hcp (hcp = “hexagonal close-packed”; refers to hollow sites with a gold atom in the layer below the upper one) or hollow fcc (fcc = “face-centered cubic”; refers to hollow sites without any atom in the layer below the upper one).

Adsorption is allowed on hcp sites that are unoccupied, as well as the three neighboring fcc sites, and on unoccupied fcc sites together with their hcp neighboring sites. Adsorption rates are considered constant for each simulation and independent of the environment. These values are parameterized.

Diffusion rates are allowed only between one hcp site and one of its three neighboring fcc sites, and vice versa. The diffusion velocities depend on the different environments (occupation of neighboring sites by other Pt atoms).

### 2.2. Kinetic Monte Carlo Method

**Figure 1:**
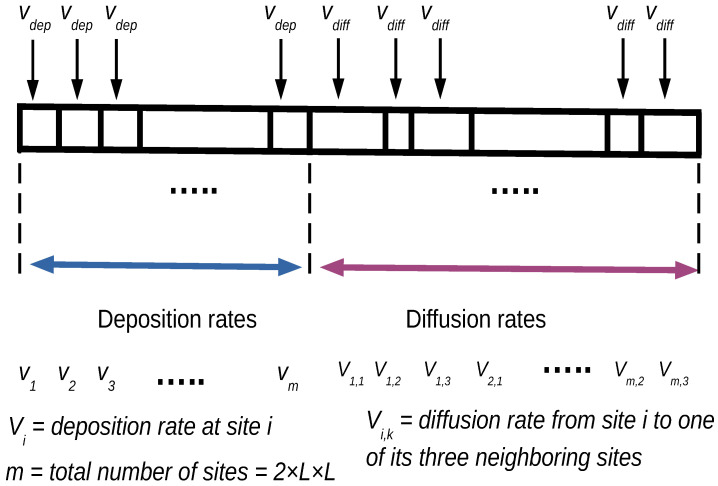
Schematic representation of the different rates for each possible process that can occur at each KMC step. We have m=2×L×L of deposition rates, and they can take only two possible values: $v_{dep}$ or 0 depending on whether the site and its neighboring sites are unoccupied or not. On the other hand, the possible values of $v_{diff}$ are all different, depending on the environment.

Monte Carlo methods are used as computational tools in many areas of physical chemistry. Although traditionally applied to obtain equilibrium properties, MC methods can also be used to study dynamic phenomena [43,44]. In the KMC method, every step consists of a random selection of one of many possible processes. The probability of a process being selected is directly proportional to its rate, which is represented as a fraction of a segment containing all processes that may occur in the system (see Figure 1). The rates of all possible processes are stored in an array of length nsites+ndiffusion, where nsites=2×L×L is the total number of surface sites (where each component of the array is associated with a rate equal to $v_{dep}$ if the site is available or 0 if it is not, i.e., when the site or one of its three neighboring sites is occupied), and ndiffusion=3×nsites is the total number of possible diffusion movements from each site to one of its three neighboring sites (where each component of the array is associated with a rate equal to $v_{dif}$—different for each environment—if there is a particle that can diffuse or 0 otherwise). Using a random number, the next process to occur is selected with a probability proportional to its rate.

**Figure 2:**
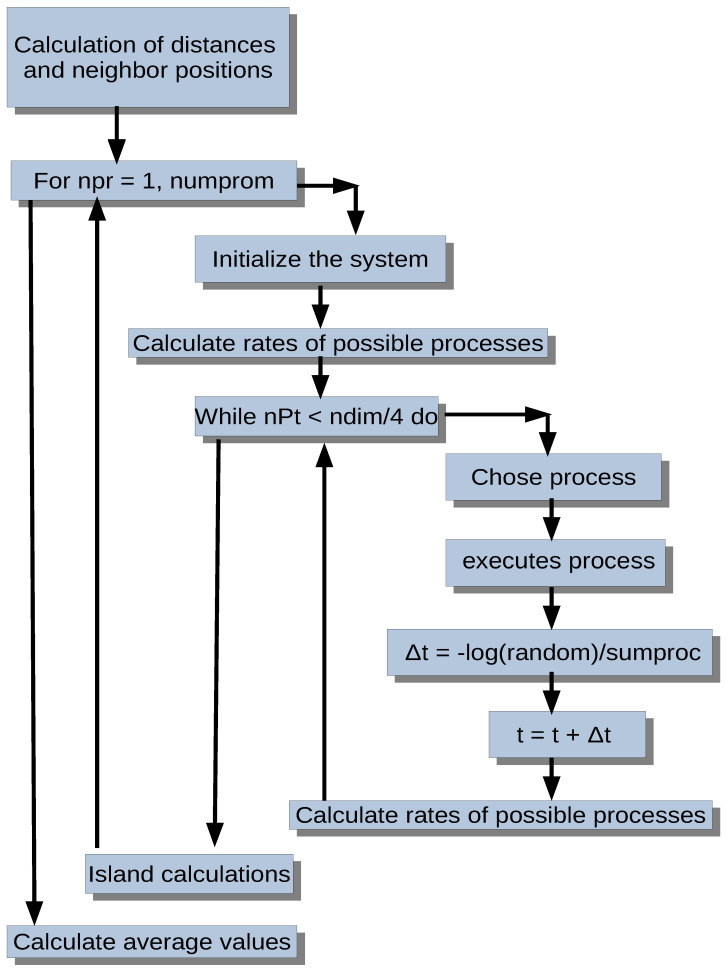
Flowchart of the simulation program. Here, npr is the number of simulation runs, numprom is the total number of simulations averaged, nPt is the number of Pt atoms, ndim is the system size (=L×L), sumproc is the sum of the rates of all possible processes ($=\sum_{i}v_{i}$), and random is a random number, uniformly distributed in [0,1). The random number generator employed is the ran2() subroutine from Numerical Recipes.

Once the randomly chosen process has occurred, all possible processes are calculated and stored again in the corresponding fraction of the segment, and a time increment of $\Delta t=-ln\left(u\right)/\sum v_{i}$ is added, where *u* is a random number between 0 and 1, and $\sum v_{i}$ is the sum of the rates of all possible processes. This choice of the time increment is due to the assumption that we are dealing with a Poisson process [42,43,44]. A general flowchart of the employed algorithm is shown in Figure 2.

**Figure 3:**
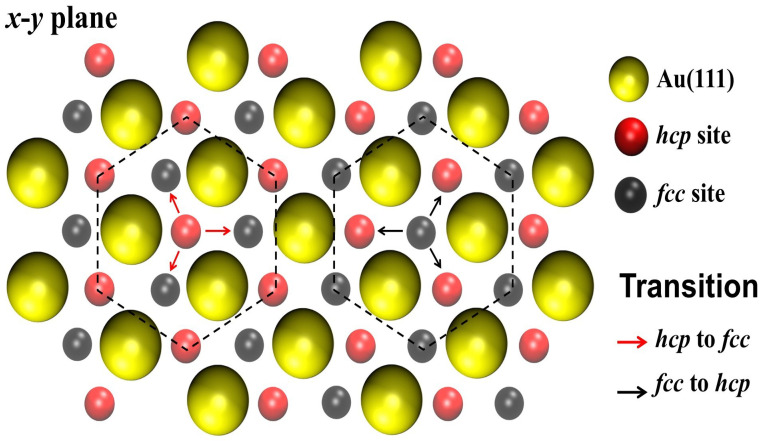
Portion of the supercell used to perform the present simulations, showing the position of atoms on the lattice. Large yellow circles: gold atoms from the upper plane of the (111) surface. Small gray circles: fcc hollow adsorption sites. Small pink circles: hcp hollow adsorption sites. The fcc and hcp hollow sites correspond to the plane of the adsorbates. An adsorbed Pt atom can diffuse from an fcc site to one of the three neighboring hcp sites, and vice versa.

To model the (111) gold surface and the possible adsorption sites for Pt atoms, a lattice containing three types of sites on the (111) surface was generated. One type of site corresponds to the positions of the Au atoms of the substrate. The other two types of sites correspond to the possible positions at which adatoms can be adsorbed, that is, at fcc and hcp sites. The corresponding geometry is shown in Figure 3, where the positions of the Au atoms of the surface are also shown for clarity.

We say that a system is of size *L* when it is made of a plane that has L×L fcc and L×L hcp adsorption sites. The values of *L* analyzed here were 10, 20, 30, 40, 50, 60, 70, and 80. The adsorption of atoms is restricted in such a way that when a Pt atom is adsorbed at an fcc (hcp) site, none of its three neighboring hcp (fcc) sites can be simultaneously occupied. This restriction is schematically depicted in Figure 3: the red arrows show the Pt adsorption at the hcp site, its possible diffusion toward one of the three neighboring fcc sites, and the blocking of adjacent hcp sites; the black arrows show the Pt adsorption at fcc sites.

For the diffusion of adatoms, the restriction of their motion is such that one atom adsorbed at an fcc (hpc) site can only diffuse toward one of its three hcp (fcc) neighboring sites (see Figure 3), with the condition that the final site and its corresponding neighbors (of the other type) must be empty to allow the move.

At each KMC step, two possible processes may occur: diffusion of an atom toward one of its neighboring sites if it is empty or adsorption of a Pt atom. Thus, the adsorption process is considered irreversible. Surface alloying is not considered in the present approach, but it could be included in future formulations.

### 2.3. Energy Calculation

To calculate the velocities for adatom diffusion in different environments, a simple model based on embedded-atom method (EAM) calculations was used [47]. This method takes into account many-body effects; therefore, it represents the metallic bonding better than a pair potential. El-koraychy et al. [48] found that EAM and DFT results are in very good agreement, validating the embedded-atom approach for heteroepitaxial growth.

The EAM is a semi-empirical method that considers the total energy $U_{tot}$ of an arrangement of *N* particles, calculated as the sum of energies $U_{i}$ corresponding to individual particles:

(3) $$U_{tot}=\sum_{i=1}^{N}U_{i}$$

where $U_{i}$ is given by

(4) $$U_{i}=F_{i}\left(\rho_{h,i}\right)+\frac{1}{2}\sum_{j\neq i}V_{ij}\left(r_{ij}\right)$$

where $F_{i}$ is called the embedding function and represents the energy necessary to embed atom *i* in the electronic density $\rho_{h,i}$ at the position at which this atom is located. The repulsion between ion cores is represented through a pair potential $V_{ij}\left(r_{ij}\right)$, which depends on the distance between the cores $r_{ij}$.

We employed the EAM within a lattice model, which is described in more detail in [31,32,33,34].

### 2.4. Diffusion Rate Calculations

The diffusion rates used in the simulation were calculated according to the different environments that an atom may encounter on the surface. Various configurations were taken into account according to the different possible arrangements of atoms near the starting and ending sites. Prior to the KMC simulation, for several configurations, the path followed by an atom to jump from one site to a neighboring one was traced, and the energy in each position was calculated, minimizing it with respect to z (the coordinate perpendicular to the surface plane). The activation energy, $E_{a}$, was calculated as the difference between the saddle point and the initial minimum in the energy curve along the reaction coordinate. The vibrational frequency ν of the atom at the starting site was calculated by performing the harmonic approximation near the minimum of the curve without any neighbors. More details of this calculation procedure can be found in [33,34].

For each individual diffusion process, we considered that one atom positioned at a particular lattice site had three possibilities for moving toward one of the three nearest neighboring sites corresponding to the other sublattice (if the original position is on an fcc site, it can diffuse toward an hcp site, and vice versa). For each possible movement, eight neighboring sites were taken into account, four corresponding to the initial site and four corresponding to the final site. For each sublattice, every site has six nearest neighbors of the same sublattice, but the two closest to the other site must be unoccupied for the diffusion to be possible. Each neighboring site can have two possible occupation states: 0 (unoccupied) or 1 (occupied by a Pt atom), totaling $2^{8}$ possible configurations, with each configuration corresponding to a particular value of the diffusion velocity.

**Figure 4:**
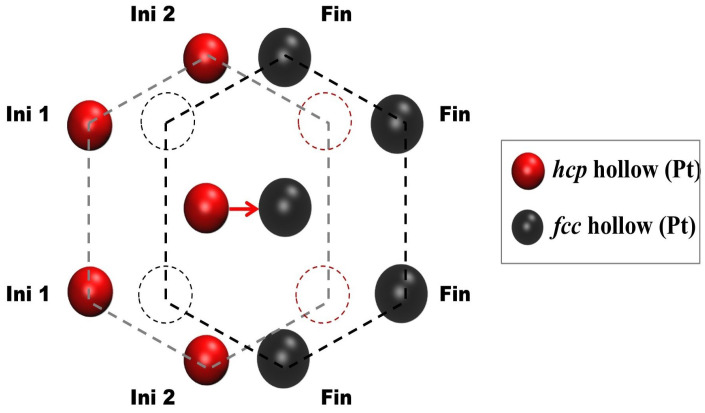
Schematic representation of the diffusion of a Pt atom from one site to a neighboring one. If the initial site is of the fcc type, the sites labeled Ini1 and Ini2 are as well, and the final site, together with the site labeled Fin, is of the hcp type. On the other hand, if the initial site is of the hcp type, the sites labeled Ini1 and Ini2 are as well, and the final site, together with the site labeled Fin, is of the fcc type. The eight surrounding sites are taken into account for the calculation of the diffusion velocity. The four sites closest to the initial and final sites must be empty due to steric hindrance.

Figure 4 shows a schematic representation of the diffusion process for a Pt atom from one type of site to a neighboring site of the other type. The eight surrounding sites taken into account in the calculation of the velocity are shown and differentiated with the following labels: Ini1 and Ini2 for sites next to the initial site (which are of the same type as the initial site, either fcc or hcp) and Fin for the four sites next to the final site (which are of the same type as the final site and opposite to the type of the initial site).

In order to calculate the diffusion velocities, the following considerations were made:

1. The activation energy in the absence of any neighbors was assumed to be 0.086 eV. This corresponds to the value obtained from the EAM calculation for the motion of a single Pt atom between two neighboring adsorption sites on a Au(111) surface in the absence of any neighbors.
2. The vibrational frequency ν of the atom at the starting site in the absence of any neighbors was $0.87\times 10^{12}$ s^−1^, obtained from the same EAM calculations. The assumption here was that changes in ν due to the presence of neighboring atoms are negligible compared with changes in the activation energy. So, we considered the same vibrational frequency for all possible configurations.
3. The fastest diffusion process was assumed to be the diffusion from a site without neighbors to a site surrounded by 4 Pt atoms. The activation energy for this process was set to zero.
4. Two types of contributions to the activation energy were assumed to arise when the neighboring sites of the initial site are occupied, say $\Delta E_{Pt-Ini1}$ and $\Delta E_{Pt-Ini2}$. These were obtained from EAM calculations, yielding $\Delta E_{Pt-Ini1}=1.10$ eV/atom and $\Delta E_{Pt-Ini2}=0.17$ eV/atom.

Thus, the activation energy is calculated as

(5) $$E_{a}=E_{act-0}+n_{Pt-Ini1}\Delta E_{Pt-Ini1}+n_{Pt-Ini2}\Delta E_{Pt-Ini2}+n_{Pt-Fin}\Delta E_{Pt-Fin}$$

where $n_{Pt-Ini1}$ is the number of Pt atoms adsorbed on the two type-1 neighboring sites of the initial site (those sites located farther away from the arriving site), $n_{Pt-Ini2}$ is the number of Pt atoms adsorbed on the two type-2 neighboring sites of the initial site (those sites located closer to the arriving site), and $n_{Pt-Fin}$ is the number of Pt atoms adsorbed on the four neighboring sites of the final site.

Taking into account the parameters indicated in Table 1 and the activation energy calculated according to Equation (5), the velocity for each possible diffusion event was calculated as $v=\nu \;exp(-E_{a}/kT)$. Here, the considered temperature was T=300 K, so kT≈0.026 eV.

The parameters listed in Table 1 were selected according to the method described in the first paragraph of this section for some particular configurations. In particular, the values of ν and $E_{act-0}$ correspond to the case of diffusion of one single Pt atom on the surface in the absence of nearest neighbors. For the value of the parameter $E_{Pt-Ini1}$, the activation energy corresponding to the configuration with two nearest neighbors at type-1 initial sites was taken into account (the value of the parameter was calculated as $E_{Pt-Ini1}=\left(E_{act}-E_{act-0}\right)/2$). For $E_{Pt-Ini2}$, the procedure was the same, but for type-2 sites. On the other hand, the parameter $E_{Pt-Fin}$ was calculated as $E_{Pt-Fin}=-E_{act-0}/4$ in order to obtain the maximum possible value of velocity (that with $E_{a}=0$ eV) in the case of four nearest neighbors next to the arriving site and none next to the starting site.

### 2.5. Deposition Rates

Let us consider $Pt^{4+}$ ions in the electrolytic solution, generally complexed as ${\left(PtCl_{6}\right)}^{2-}$, with a constant activity ($a_{Me^{z+}}$=$a_{Pt^{4+}}$) at a temperature T. The electrochemical deposition/dissolution rates at a given electrode potential (E), measured against a reference electrode, are given by the following equations:

(6) $$v_{dep}\left(E\right)=k_{dep}^{0}a_{Me^{z+}}exp\left(-\frac{\Delta G_{dep}^{\#(0)}}{k_{B}T}\right)exp\left(-\frac{(1-\alpha )zFE}{k_{B}T}\right)$$

(7) $$v_{diss}\left(E\right)=k_{diss}^{0}exp\left(-\frac{\Delta G_{diss}^{\#(0)}}{k_{B}T}\right)exp\left(\frac{\alpha zFE}{k_{B}T}\right)$$

where the values of $\Delta G^{\#(0)}$ are the activation energies for the ion transfer from the solution to the crystal, or vice versa, at E=0; α is the charge transfer coefficient; $a_{Me^{z+}}$ is the activity of metal ions in the electrolyte; and $k_{dep}^{0}$ and $k_{diss}^{0}$ are the rate constants for the deposition and the dissolution reaction at each site [1,34].

The above equations indicate that each site is subject to an equilibrium between deposition and dissolution. This equilibrium can be altered by the electrode potential. It should be noted that the simulations were performed at potentials where $v_{dep}\left(E\right)>>v_{diss}\left(E\right)$. According to Equations (6) and (7), this occurs because the exponential term in the electric potential in the reaction is the dominant one. Under these conditions, one can consider $v_{diss}\left(E\right)=0$.

In the present work, we considered different values of $v_{dep}=v_{dep}\left(E\right)$ ($=1\times 10^{n}\;$s^−1^, with n=0,1,2,3,...,12). These values correspond to different possible electrode potentials, *E*. The dissolution rate $v_{diss}$ was neglected. Thus, in the present model, each site was subject to two types of movements: diffusional, where the (surface) diffusion rates were calculated depending on the environment, and deposition (or adsorption), whose rate was introduced parametrically.

## 3. Results

### 3.1. Diffusion Rates

**Figure 5:**
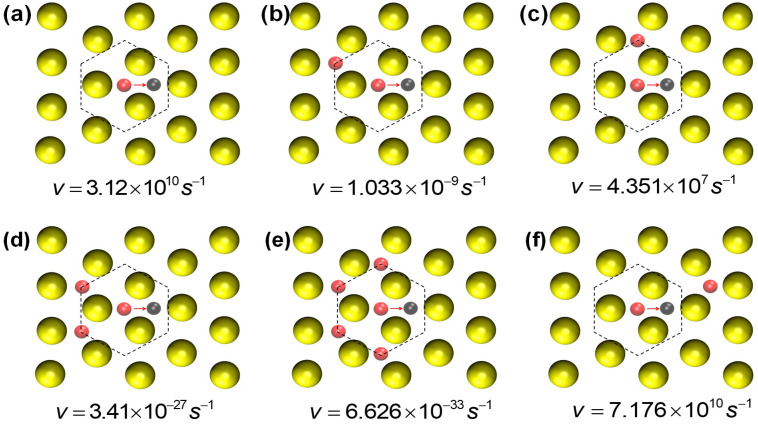
Some possible configurations for the diffusion of a Pt atom and the corresponding rates. Large yellow circles: gold atoms from the upper plane of the 111 surface. Small pink circles: Pt atoms on the surface (the central one, for diffusion, and the surrounding ones). The arrow indicates the direction of the Pt atom displacement. (**a**) Difusion without neighbors. (**b**) With one neighbor in a site of kind $Ini_{1}$. (**c**) With one neighbor in a site of kind $Ini_{2}$. (**d**) With two neighbors at sites of kind $Ini_{1}$. (**e**) With two neighbors at sites of kind $Ini_{1}$ and two at sites of kind $Ini_{2}$. (**f**) With one neighbor in a site of kind Fin.

Figure 5 shows the rate values for the diffusion of a Pt atom on the surface, with six possible environments. It can be seen that the diffusion rate on a clean surface is on the order of $10^{10}\;$s^−1^, whereas a nearest-neighboring Pt atom located close to the initial state of the movement decreases the probability of diffusion to a rate on the order of $10^{-9}\;$s^−1^ or $10^{7}\;$s^−1^, depending on the location of the neighbor. On the other hand, a Pt atom located near the site of arrival increases the diffusion rate to a rate of $7\times 10^{10}\;$s^−1^. From configurations (b), (d), and (e) in Figure 5, we learn that the detachment of a Pt atom from a Pt cluster is almost impossible.

### 3.2. Pt Deposition on Au(111)

The formation of Pt 2D clusters on Au(111) surfaces was analyzed at different deposition rates on systems of different sizes L×L, corresponding to substrates with supercells containing between 100 (L=10) and 6400 (L=80) atoms. Although the system was, in principle, infinite, since the simulation supercell was repeated with periodic boundary conditions in two dimensions (*x* and *y*), finite-size effects appeared, since the largest adsorbate island cannot be larger than the dimension of the supercell employed. We analyze this problem in the following paragraphs. Since the diffusion rates were fixed due to the fact that they were all calculated based on selected parameters, the free variable taken into account was the deposition rate $v_{dep}$, which is the rate assigned to the adsorption of a single Pt atom from the solution and was taken as a parameter to explore the behavior of the system. The values considered were $v_{dep}=10^{n}\;$s^−1^, with n=0,1,2,...,12. Although larger $v_{dep}$ values are physically unrealistic because they correspond to very large experimental overpotentials, they were included anyway to provide a view of the velocity ranges where different behaviors may be expected.

#### 3.2.1. Time Evolution

**Figure 6:**
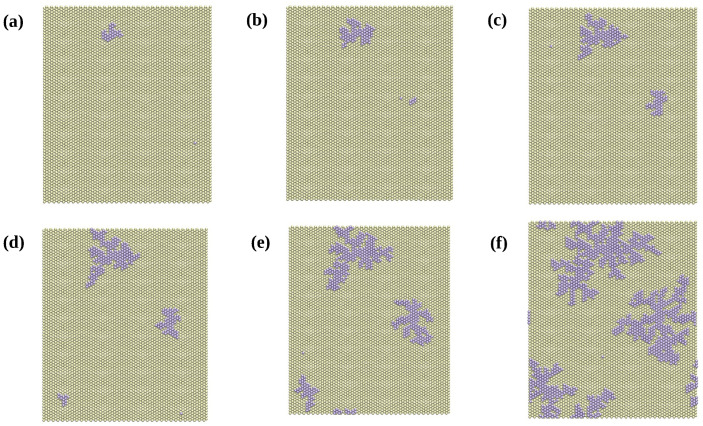
KMC simulation of the deposition of Pt on Au(111) for L=70 and $v_{dep}=10^{2}\;$s^−1^. Frames show the morphology of the submonolayer adsorbed on the surface at different times. Yellow: gold atoms. Violet: platinum atoms. (**a**) t=39.9μs. (**b**) t=68.6μs. (**c**) t=128.5μs. (**d**) t=263.2μs. (**e**) t=521.8μs. (**f**) t=1479.2μs.

Figure 6 shows snapshots of the simulation for platinum adsorption for a system size L=70 and a deposition rate $v_{dep}=10^{2}$ s^−1^, corresponding to different stages of the KMC simulation at different times, as indicated. It can be observed that a few islands with dendritic-type structures formed for a final deposition time on the order of milliseconds. The shape of the islands can be explained by the fact that free atoms on the surface can diffuse until they reach an existing island, and then diffusion around the steps or leaving the island becomes very difficult due to the low velocities associated with these processes.

**Figure 7:**
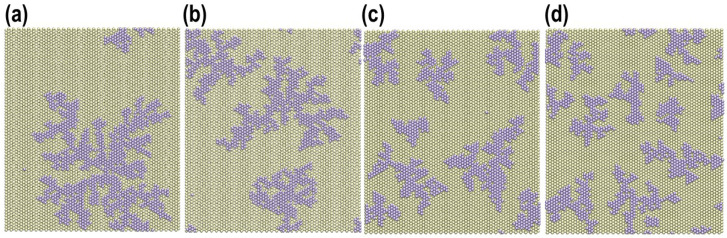
KMC simulation of the deposition of Pt on Au(111) for L=70 and different deposition rates $v_{dep}$. Frames show the final state of the simulation for the coverage degree θ=0.25. Yellow: gold atoms. Violet: platinum atoms. (**a**) $v_{dep}=10^{1}\;$s^−1^. (**b**) $v_{dep}=10^{2}\;$s^−1^. (**c**) $v_{dep}=10^{3}\;$s^−1^. (**d**) $v_{dep}=10^{4}\;$s^−1^.

On the other hand, Figure 7 shows snapshots of the final states of the simulations for platinum adsorption for a system size L=70 and deposition rates $v_{dep}=10^{1}\;$s^−1^, $v_{dep}=10^{2}\;$s^−1^, $v_{dep}=10^{3}\;$s^−1^, and $v_{dep}=10^{4}\;$s^−1^. For the lowest deposition rate, only one island can be observed, while for higher deposition rates, several small islands can be seen.

Here, the average island size is defined as

(8) $$<S>=\frac{\sum_{j=1}^{N_{i}}S\left(j\right)}{N_{i}}$$

where $N_{i}$ is the total number of islands (including monomers) and S(j) is the size (number of atoms) of the *j*-th island.

**Figure 8:**
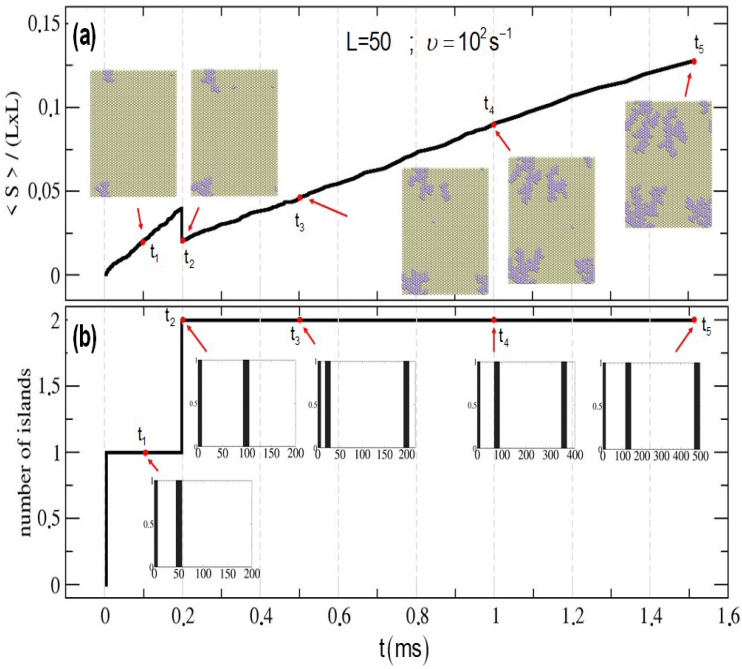
Time evolution of the deposition of Pt on Au(111). (**a**) Average island size (normalized by the system size). Snapshots of the surface at different times are included. (**b**) Number of islands. Histograms corresponding to the island distribution are also included. Deposition velocity $v_{dep}=10^{2}$. System size L=50.

**Figure 9:**
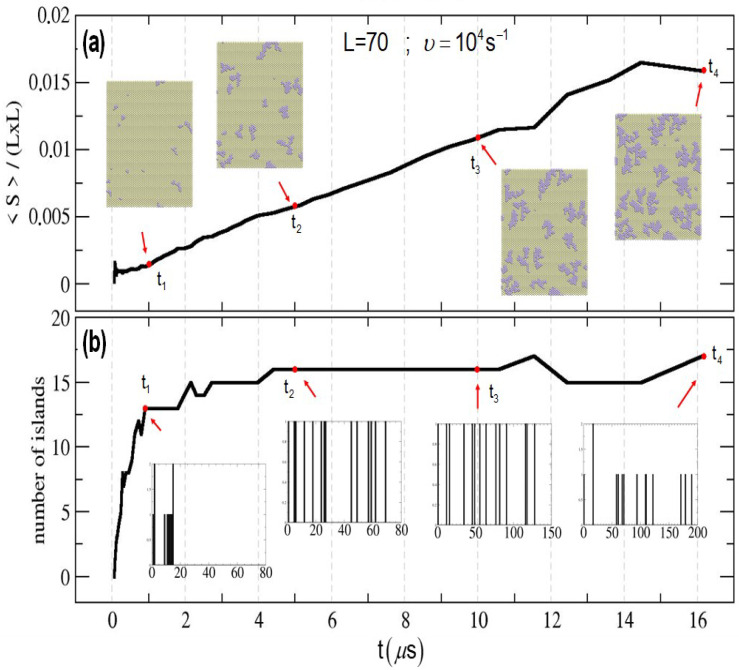
Time evolution of the deposition of Pt on Au(111). (**a**) Average island size (normalized by the system size). Snapshots of the surface at different times are included. (**b**) Number of islands. Histograms corresponding to the island distribution are also included. Deposition velocity $v_{dep}=10^{4}$. System size L=70.

Figure 8 and Figure 9 show the time evolution of the average island size (normalized by the system size) and the total number of islands for two cases used as examples: a system size L=50 and a deposition rate $v_{dep}=10^{2}\;$s^−1^ (Figure 8), and a system size L=70 and a deposition rate $v_{dep}=10^{4}$ (Figure 9). Snapshots of the state of the surface and histograms for the island distribution are also included for selected times.

For the simulation shown in Figure 8, only one island is present until $t\approx 2\times 10^{-4}$ s. At that particular time, a second island is formed. So, the average island size increases nearly linearly until $t\approx 2\times 10^{-4}$ s, then decreases abruptly due to the formation of a second island, and subsequently continues to increase linearly but with a lower slope compared to the initial stage.

For the simulation shown in Figure 9, as the system size is larger and the deposition rate is higher, a larger number of smaller islands than in the previous example is observed. The total number of islands initially increases, corresponding to a nucleation and growth stage, and for times around $t\approx 4\times 10^{-6}$ s, this number remains more or less constant, corresponding to a growth stage (the existing islands continue growing in size, but no new islands are formed).

#### 3.2.2. Average Values in Final States

**Figure 10:**
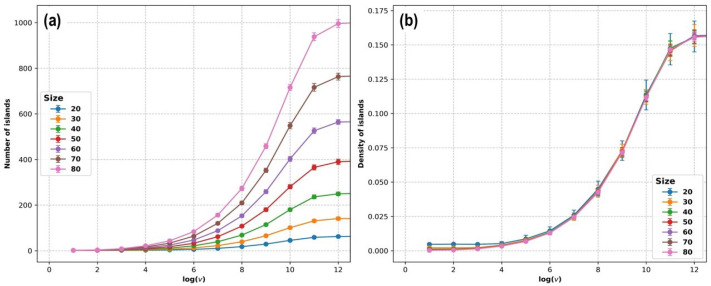
KMC simulations of the deposition of Pt on Au(111) until reaching a coverage of 0.25. (**a**) The total number of islands in the final state is plotted as a function of the logarithm of the deposition rate $v_{dep}$. (**b**) The density of islands (number of islands normalized by the system size) in the final state is plotted as a function of the logarithm of the deposition rate $v_{dep}$. Each point corresponds to the average over 100 simulations. Error bars are shown as vertical segments for each point.

**Figure 11:**
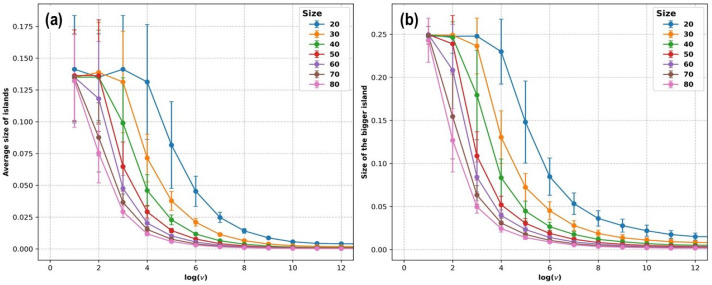
KMC simulations of the deposition of Pt on Au(111) until reaching a coverage of 0.25. (**a**) The average size of islands (normalized by the system size) in the final state is plotted as a function of the logarithm of the deposition rate $v_{dep}$. (**b**) The size of the bigger island (normalized by the system size) in the final state is plotted as a function of the logarithm of the deposition rate $v_{dep}$. Each point corresponds to the average over 100 simulations. Error bars are shown as vertical segments for each point.

Figure 10 and Figure 11 show the total number of islands, the density of islands, the average size of islands, and the size of the biggest island in the final state (which corresponds to θ=0.25; the simulation ends when the last particle necessary to reach this coverage degree adsorbs) as a function of the logarithm of the deposition rate $v_{dep}$, for system sizes corresponding to L=10,20,30,40,50,60,70, and 80, with each case averaged over 100 KMC simulations.

In Figure 10a, it can be seen that the total number of islands increases with the deposition rate. The system size also influences the number of islands. But, as can be seen in Figure 10b, when the island density is considered (the number of islands normalized by the system size), the different curves overlap, except for the case when L=10 (and to a lesser extent for L=20), where the density is higher than that observed for the other system sizes. This shows that finite-size effects are only evident for the smallest system sizes but are negligible for most of the system sizes considered here.

Figure 11 shows the results obtained from the simulations for the average island size <s> and the maximum island size $<s_{max}>$, normalized by the size of the simulation box, $L^{2}$. Both plots show qualitatively similar behavior, with a plateau at lower values of $v_{dep}$, followed by a decrease, and finally asymptotic behavior toward a small value for very high rates. The plateau at lower values of $v_{dep}$ is a result of the finite-size effect: if the deposition rate is low enough, a single island covers the whole simulation supercell, and the physical relevance of the simulation is lost. The value $<s_{max}>\approx 0.25$ for low values of $v_{dep}$ indicates a single large island with a size almost given by the coverage degree, while a smaller value of <s> for lower values of $v_{dep}$ (Figure 11a) indicates the presence of a few small islands or even single atoms, which are not shown in Figure 11b.

**Figure 12:**
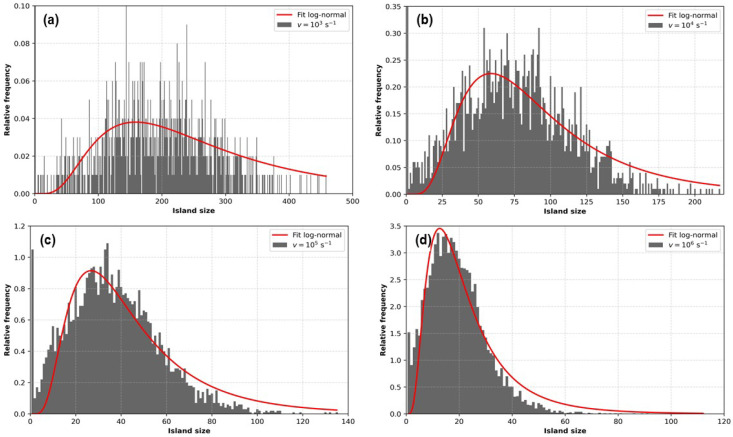
Histograms corresponding to the island size distribution in the final state for four values of the deposition rate: (**a**) $v_{dep}=10^{3}$ s^−1^, (**b**) $v_{dep}=10^{4}$ s^−1^, (**c**) $v_{dep}=,10^{5}$ s^−1^, and (**d**) $v_{dep}=10^{6}$ s^−1^. Each histogram corresponds to the average over 100 KMC simulations. System size L=80. The red curves correspond to a log-normal fit (see the discussion in the text and Equation (9)).

Figure 12 shows histograms for the island size distribution at the final state for four different deposition rates. In these cases, the system size is L=80, and 100 KMC simulations are averaged. As can be seen, for low deposition rates, the size distribution of islands becomes wide, and the island sizes reach relatively large values. As the deposition rate increases, the size distribution becomes narrower, and the mean shifts toward lower values.

**Figure 13:**
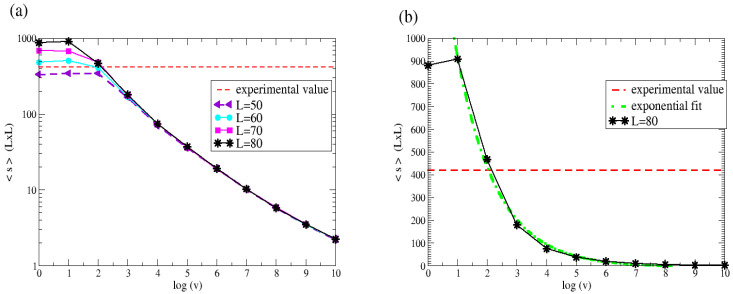
KMC simulations of the deposition of *Pt* on *Au*(111) until reaching a coverage of *θ* = 0.25. The average size of islands in the final state is plotted as a function of the logarithm of the deposition rate *v_dep_*. Each point corresponds to the average over 100 simulations. Red dashed lines show the experimental values estimated at < *s* > ≈ 420. In (**a**), the four largest system sizes are considered, and a logarithmic scale is employed on the y-axis. In (**b**), only the system size *L* = 80 is shown, together with an exponential fit of the data.

Figure 13 shows a plot of the average island sizes (the same cases as in Figure 11a, but multiplied by L×L), together with the value corresponding to the experimental case. In Figure 13a, the four largest system sizes are shown together on a logarithmic scale on the y-axis, and it can be seen that, except for the lower values of $v_{dep}$, the system size effects are negligible.

**Figure 14:**
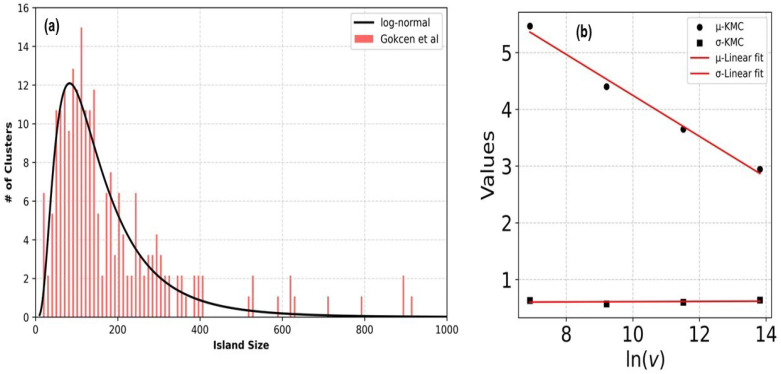
(**a**) Histogram of the Pt-on-Au cluster population, adapted from [18]. (**b**) Relationships between the parameters σ and μ obtained from the log-normal fit of the KMC simulations and the logarithm of the deposition rate. The parameter values fitted for the experimental size distribution are μ=4.8892 and σ=0.6862.

Concerning the comparison with the experiments, the theoretical histograms in Figure 12 show remarkable features, which were compared with those of similar experimental results from Ref. [18]. These results are reproduced in Figure 14. In this figure, we see that the shape of the distribution of the clusters obtained in [18], depicted in red bars, strongly resembles the distribution curves in Figure 12.

To seek a more quantitative comparison, we employ a lognormal distribution function used in the literature to describe the distribution of nanocrystal clusters [49,50]:

(9) $$f\left(S\right)=\frac{1}{S\sigma \sqrt{2\pi }}exp\left(-\frac{{\left(ln\left(S\right)-\mu \right)}^{2}}{2\sigma^{2}}\right)\;for\;S>0$$

where *S* represents the island size, μ is the mean value of the logarithm of the island sizes, and σ is the standard deviation. The red curves in Figure 12 correspond to the fitting of the numerical data to Equation (9), and the parameters μ and σ are given in Table 2.

These parameters are represented as a function of the logarithm of the deposition rate $v_{dep}$ in the inset of Figure 14. It can be seen that while σ is insensitive to $v_{dep}$, μ shows a linear dependence on $ln(v_{dep})$. When the same fitting is applied to the experimental data from Figure 14, we obtain μ=4.8892 and σ=0.6862. Thus, we find remarkable agreement between the σ of the experimental distribution and the theoretical values reported in Table 2, indicating that the simulations captured the physical features of the experiment.

## 4. Conclusions

Kinetic Monte Carlo simulations for the deposition and diffusion of Pt atoms on Au(111) surfaces were performed to study the electrochemical deposition and spontaneous island formation of platinum on gold. On one hand, the diffusion rates were calculated using absolute rate theory, with some parameters based on EAM calculations. On the other hand, the deposition rates were treated as a parameter that could take several possible values (emulating different experimental conditions).

The time evolution and final states for the coverage degree θ=0.25 were studied for different values of $v_{dep}$. In all cases, the morphology of the deposited island was found to be rather ramified, instead of forming compact islands. The density of islands at the final state (for θ=0.25) increased with increasing $v_{dep}$, and the curves were found to be independent of the system size, except for L=10 (very small). The average island size (normalized by system size) as a function of $v_{dep}$ was approximately constant for low deposition velocities and then decreased as $v_{dep}$ increased, depending on the system size. The island size distribution at the final state was broader and tended toward higher values at lower $v_{dep}$.

## Figures and Tables

**Table 1:** Parameters employed for the calculation of diffusion rates.

Parameter	Value
ν (frequency)	$0.87\times 10^{12}$ s^−1^
$E_{act-0}$	0.086 eV
$\Delta E_{Pt-Ini1}$	1.10 eV
$\Delta E_{Pt-Ini2}$	0.17 eV
$\Delta E_{Pt-Fin}$	−0.0215 eV

**Table 2:** Parameters of the log-normal fit of the island size histograms shown in Figure 12a–d, using Equation (9).

$v_{\mathrm{dep}}$ (s^−1^)	μ	σ
$10^{3}$	5.4651	0.6320
$10^{4}$	4.3992	0.5713
$10^{5}$	3.6485	0.6005
$10^{6}$	2.9452	0.6395

## Data Availability

The raw data supporting the conclusions of this article will be made available by the authors on request.

## Abbreviations

The following abbreviations are used in this manuscript:

MC	Monte Carlo
MD	Molecular Dynamics
NPs	Nanoparticles
STM	Scanning Tunneling Microscope
KMC	Kinetic Monte Carlo
GCMC	Grand Canonical Monte Carlo
UPD	Underpotential Deposition
OPD	Overpotential Deposition
ED	Electrochemical deposition
SD	Spontaneous Deposition
GR	Galvanic Replacement
SLRR	Surface-Limited Redox Replacement
EAM	Embedded-Atom Method
DFT	Density Functional Theory
fcc	face-centered cubic
hcp	hexagonal close-packed

## References

[1] Budevski E., Staikov G., Lorenz W.J. (1996). Electrochemical Phase Formation and Growth.

[2] Staikov G., Lorenz W.J., Budevski E., Ross P., Lipkowski J. (1999). Imaging of Surfaces and Interfaces—Frontiers of Electrochemistry.

[3] Oviedo O.A., Reinaudi L., Garcia S., Leiva E.P.M. (2016). Underpotential Deposition, From Fundamentals and Theory to Applications at the Nanoscale.

[4] Brankovic S. (2014). Electrocatalysis: Novel Synthetic Methods in Electrocatalysis: Novel Synthetic Methods.

[5] Kowal A., Li M., Shao M., Sasaki K., Vukmirovic M.B., Zhang J., Marinkovic N.S., Liu P., Frenkel A.I., Adzic R.R. (2009). Ternary *Pt*/*Rh*/*SnO*_2_ electrocatalysts for oxidizing ethanol to *CO*_2_. Nat. Mater..

[6] Adzic R., Zhang J., Sasaki K., Vukmirovic M.B., Shao M., Wang J.X., Nilekar A.U., Mavrikakis M., Valerio J.A., Uribe F. (2007). Platinum monolayer fuel cell electrocatalysts. Top. Catal..

[7] Sasaki K., Wang J.X., Naohara H., Marinkovic N., More K., Inada H., Adzic R.R. (2010). Recent advances in platinum monolayer electrocatalysts for oxygen reduction reaction: Scale-up synthesis, structure and activity of *Pt* shells on *Pd* cores. Electrochim. Acta.

[8] Gokcen D., Yuan Q., Brankovic S.R. (2014). Nucleation of *Pt* monolayers deposited via surface limited redox replacement reaction. J. Electrochem. Soc..

[9] Gokcen D., Bae S.E., Mohammedy P., Liu P., Brankovic S.R. (2012). Size effects in monolayer catalysis-model study: *Pt* submonolayers on *Au*(111). Electrocatalysis.

[10] Waibel H.-F., Kleinert M., Kibler L.A., Kolb D.M. (2002). Initial stages of *Pt* deposition on *Au*(111) and *Au*(100). Electrochim. Acta.

[11] Venables J.A., Spiller G.D.T., Hanbücken M. (1984). Nucleation and growth of thin films. Rep. Prog. Phys..

[12] Bakos I., Szabó S., Pajkossy T. (2011). Deposition of platinum monolayers on gold. J. Solid State Electrochem..

[13] Brankovic S.R., Breen J.M., Adžic R.R. (2001). *Pt* submonolayers on metal nanoparticles-novel electrocatalysts for *H*_2_ oxidation and *O*_2_ reduction. Surf. Sci..

[14] Uosaki K., Ye S., Naohara H., Oda Y., Haba T., Kondo T. (1997). Electrochemical epitaxial growth of a *Pt*(111) phase on an *Au*(111) electrode. J. Phys. Chem. B.

[15] Karali T., Ölmez S., Yener G. (1996). Study of spontaneous deposition of 210Po on various metals and application for activity assessment in cigarette smoke. Appl. Radiat. Isot..

[16] Strbac S., Petrovic S., Vasilic R., Kovacc J., Zalar A., Rakocevic Z. (2007). Carbon monoxide oxidation on *Au*(111) surface decorated by spontaneously deposited *Pt*. Electrochim. Acta.

[17] Kim J., Jung C., Rhee C.K., Lim T.-H. (2007). Electrocatalytic oxidation of formic acid and methanol on *Pt* deposits on *Au*(111). Langmuir.

[18] Gokcen D., Bae S.-E., Brankovic S.R. (2010). Stoichiometry of *Pt* submonolayer deposition via surface-limited redox replacement reaction. J. Electrochem. Soc..

[19] Gokcen D., Bae S.-E., Brankovic S.R. (2011). Reaction kinetics of metal deposition via surface limited red-ox replacement of underpotentially deposited metal monolayers. Electrochim. Acta.

[20] Nagahara Y., Hara M., Yoshimoto S., Inukai J., Yau S.L., Itaya K. (2004). In situ scanning tunneling microscopy examination of molecular adlayers of haloplatinate complexes and electrochemically produced platinum nanoparticles on *Au*(111). J. Phys. Chem. B.

[21] Brankovic S.R., Marinkovic N.S., Wang J.X., Adzic R.R. (2002). Electrosorption and catalytic properties of bare and *Pt* modified single crystal and nanostructured *Ru* surfaces. J. Electroanal. Chem..

[22] Nutariya J., Fayette M., Dimitrov N., Vasiljevic N. (2013). Growth of *Pt* by surface limited redox replacement of underpotentially deposited hydrogen. Electrochim. Acta.

[23] Patra S., Das J., Yang H. (2009). Selective deposition of *Pt* on *Au* nanoparticles using hydrogen presorbed into *Au* nanoparticles during NaBH_4_ treatment. Electrochim. Acta.

[24] Dai Y., Chen S. (2015). Oxygen reduction electrocatalyst of *Pt* on *Au* nanoparticles through spontaneous deposition. Appl. Mater. Interfaces.

[25] Kim S., Jung C., Kim J., Rhee C.K., Choi S.-M., Lim T.-M. (2010). Modification of *Au* nanoparticles dispersed on carbon support using spontaneous deposition of *Pt* toward formic acid oxidation. Langmuir.

[26] Zheng F., Wong W.T., Yung K.F. (2014). Facile design of Au@Pt core-shell nanostructures: Formation of *Pt* submonolayers with tunable coverage and their applications in electrocatalysis. Nano Res..

[27] Zhang J., Sung Y., Rikvold P.A., Wieckowski A. (1996). Underpotential deposition of *Cu* on *Au*(111) in sulfate-containing electrolytes: A theoretical and experimental study. J. Chem. Phys..

[28] Del Pópolo M.G., Leiva E.P.M. (1997). Embedded atom method study of Cu deposition on Ag(111). J. Electroanal. Chem..

[29] Oviedo O.A., Rojas M.I., Leiva E.P.M. (2005). Off lattice Monte-Carlo simulations of low-dimensional surface defects and metal deposits on *Pt*(111). Electrochem. Commun..

[30] Oviedo O.A., Mayer C.E., Staikov G., Leiva E.P.M., Lorenz W.J. (2006). Low-dimensional metallic nanostructures and their electrochemical relevance: Energetics and phenomenological approach. Surf. Sci..

[31] Giménez M.C., del Pópolo M.G., Leiva E.P.M. (1999). Monte Carlo simulation for the formation and growth of low dimensionality phases during underpotential deposition of Ag on Au(100). Electrochim. Acta.

[32] Giménez M.C., Leiva E.P.M. (2003). Comparative Monte Carlo study of monolayer growth in a heteroepitaxial system in the presence of surface defects. Langmuir.

[33] Giménez M.C., Pópolo M.G.D., Leiva E.P.M., García S.G., Salinas D.R., Mayer C.E., Lorenz W.J. (2002). Theoretical Considerations of electrochemical phase formation for an ideal Frank-van der Merwe system: *Ag* on *Au*(111) and *Au*(100). J. Electrochem. Soc..

[34] Giménez M.C., del Pópolo M.G., Leiva E.P.M. (2002). Kinetic Monte Carlo Study of Electrochemical Growth in a Heteroepitaxial System. Langmuir.

[35] Brown G., Rikvold P.A., Novotny M.A., Wieckowski A. (1999). Simulated dynamics of underpotential deposition of *Cu* with sulfate on *Au*(111). J. Electrochem. Soc..

[36] Liu J., Liu C., Conway P.P. (2009). Kinetic Monte Carlo simulation of electrodeposition of polycrystalline Cu. Electrochem. Commun..

[37] Frank S., Rikvold P.A. (2006). Kinetic Monte Carlo simulations of electrodeposition: Crossover from continuous to instantaneous homogeneous nucleation within Avrami’s law. Surf. Sci..

[38] Drews T.O., Braatz R.D., Alkire R.C. (2007). Monte Carlo simulation of kinetically limited electrodeposition on a surface with metal seed clusters. Phys. Chem..

[39] Treeratanaphitak T., Pritzker M.D., Abukhdeir N.M. (2014). Kinetic Monte Carlo simulation of electrodeposition using the embedded-atom method. Electrochim. Acta.

[40] Treeratanaphitak T., Pritzker M.D., Abukhdeir N.M. (2014). Atomistic kinetic Monte Carlo simulations of polycrystalline copper electrodeposition. Electrochem. Commun..

[41] Alonso C., Salvarezza R.C., Vara J.M., Arvia A.J., Vazquez L., Bartolome A., Baro A.M. (1990). The Evaluation of Surface Diffusion Coefficients of Gold and Platinum Atoms at Electrochemical Interfaces from Combined STM-SEM Imaging and Electrochemical Techniques. J. Electrochem. Soc..

[42] Gillespie D.T. (1976). A General Method for Numerically Simulating the Stochastic Time Evolution of Coupled Chemical Reactions. J. Comput. Phys..

[43] Fichthorn K.A., Weinberg W.H. (1989). Theoretical foundations of dynamical Monte Carlo simulations. J. Chem. Phys..

[44] Kang H.C., Weinberg W.H. (1991). Dynamic Monte Carlo with a proper energy barrier: Surface diffusion and two-dimensional domain ordering. J. Chem. Phys..

[45] Rafiee M., Bashiri H. (2019). Dynamic Monte Carlo simulations of the reaction mechanism of hydrogen production from formic acid on Ni(100). Appl. Surf. Sci..

[46] Rafiee M., Bashiri H. (2020). Catalytic decomposition of formic acid on Cu(100): Optimization and dynamic Monte Carlo simulation. Catal. Commun..

[47] Foiles S.M., Baskes M.I., Daw M.S. (1986). Embedded-atom-method functions for the fcc metals Cu, Ag, Au, Ni, Pd, Pt, and their alloys. Phys. Rev. B.

[48] El-koraychy E., Sbiaai K., Mazroui M., Boughaleb Y., Ferrando R. (2014). Numerical study of hetero-adsorption and diffusion on (100) and (110) surfaces of Cu, Ag and Au. Surf. Sci..

[49] Wang C.-R., Huang R.-B., Liu Z.-Y., Zheng L.-S. (1994). Lognormal size distributions of elemental clusters. Chem. Phys. Lett..

[50] de Lamaestre R.E., Bernas H. (2006). Significance of lognormal nanocrystal size distributions. Phys. Rev. B.

